# Preparation, Characterization and Diagnostic Valuation of Two Novel Anti-HPV16 E7 Oncoprotein Monoclonal Antibodies

**DOI:** 10.3390/v12030333

**Published:** 2020-03-19

**Authors:** Renjian Hu, Zhen Dong, Kui Zhang, Guangzhao Pan, Chongyang Li, Hongjuan Cui

**Affiliations:** 1 State Key Laboratory of Silkworm Genome Biology, Institute of Sericulture and Systems Biology, College of Biotechnology, Southwest University, Beibei, Chongqing 400716, China; hrj@cqut.edu.cn (R.H.); zdong007@swu.edu.cn (Z.D.); zhangk87@swu.edu.cn (K.Z.); m18983708534_2@163.com (G.P.); chongyangli7305@gmail.com (C.L.); 2 School of Pharmacy and Bioengineering, Chongqing University of Technology, Banan, Chongqing 400054, China; 3 Cancer Center, Medical Research Institute, Southwest University, Beibei, Chongqing 400716, China

**Keywords:** LSAB-ELISA, HPV16 E7 protein, cervical cancer, chemiluminescence immunoassay, luminol, monoclonal antibody, cancer diagnosis, human papillomavirus

## Abstract

At present, the clinical detection method of human papillomavirus (HPV) is mainly based on the PCR method. However, this method can only be used to detect HPV DNA and HPV types, and cannot be used to accurately predict cervical cancer. HPV16 E7 is an oncoprotein selectively expressed in cervical cancers. In this study, we prepared an HPV16 E7-histidine (HIS) fusion oncoprotein by using a prokaryotic expression and gained several mouse anti-HPV16 E7-HIS fusion oncoprotein monoclonal antibodies (mAbs) by using hybridoma technology. Two mAbs, 69E2 (IgG2a) and 79A11 (IgM), were identified. Immunocytochemistry, immunofluorescence, immunohistochemistry, and Western blot were used to characterize the specificity of these mAbs. The sequences of the nucleotide bases and predicted amino acids of the 69E2 and 79A11 antibodies showed that they were novel antibodies. Indirect enzyme-linked immunosorbent assay (ELISA) with overlapping peptides, indirect competitive ELISA, and 3D structural modeling showed that mAbs 69E2 and 79A11 specifically bound to the three exposed peptides of the HPV16 E7 (HPV16 E7_49–66_, HPV16 E7_73–85_, and HPV16 E7_91–97_). We used these two antibodies (79A11 as a capture antibody and 69E2 as a detection antibody) to establish a double-antibody sandwich ELISA based on a horseradish peroxidase (HRP)-labeled mAb and tetramethylbenzidine (TMB) detection system for quantitative detection of the HPV16 E7-HIS fusion oncoprotein, however, it was not ideal. Then we established a chemiluminescence immunoassay based on a labeled streptavidin-biotin (LSAB)-ELISA method and luminol detection system—this was sufficient for quantitative detection of the HPV16 E7-HIS fusion oncogenic protein in ng levels and was suitable for the detection of HPV16-positive cervical carcinoma tissues. Collectively, we obtained two novel mouse anti-HPV16 E7 oncoprotein mAbs and established an LSAB-lumino-dual-antibody sandwich ELISA method for the detection of the HPV16 E7-HIS fusion oncogenic protein, which might be a promising method for the diagnosis of HPV16-type cervical cancers in the early stage.

## 1. Introduction

The morbidity rate of cervical cancer makes it the fourth most frequent cancer in women worldwide. The high-risk human papillomavirus (HPV) integrating the cervix is a direct cause of cervical precancerous lesions and cervical cancer [1]. HPV 16 and 18 account for > 70% of cervical cancers and HPV 31, 33, 35, 45, 52, and 58 account for an additional 20% [2,3]. The prevalence of HPV infection in the cervix among women is 42.7% in the USA [4] and 17.7% in China [5]. HPV detection combined with cytology can improve the detection rate of cervical intraepithelial neoplasia (CIN) and cervical cancer [6].

At present, the clinical detection method of HPV is mainly based on the PCR method [7]. The advantages of the PCR method are that it can detect HPV DNA and HPV type, and it has high sensitivity. The disadvantage is that it cannot detect HPV E6 and E7 oncoproteins, so it cannot accurately predict cervical cancer. HPV E6 and E7 oncoproteins are selectively expressed in tumor cells and eventually appear in cervical exfoliated cells [8,9,10], so the presence of cervical E6/E7 oncoproteins can indicate the presence of cervical cancer cells. The OncoE6 ™ Cervical Test Kit currently developed by European Arbor Vita is a method based on lateral flow immunochromatography to detect E6 oncoproteins of HPV 16, 18, and 45 subtypes, which has the advantage of alerting about cervical cancer [11,12]. However, it is not sensitive and cannot be accurately quantified. During the progression of cervical carcinoma, especially in the stage of CIN, the expression of HPV E7 oncoprotein increases whereas the expression of E6 decreases [13]. Therefore, detecting E7 may be a more accurate method for the diagnosis of cervical cancers in the early stage.

In order to quantitatively detect HPV E7 oncoprotein and improve the sensitivity of HPV E7 oncoprotein detection, this project was carried out to acquire high-quality monoclonal antibodies (mAbs) and explore quantitative detection of cervical HPV16 E7 oncoproteins at the protein level based on a labeled streptavidin-biotin (LSAB)-luminol-double-antibody sandwich ELISA method. HPV16-positive cervical cancer tissue specimens were used to test the validity of this method.

## 2. Materials and Methods

### 2.1. Materials and Experimental Instruments

Human cervical cancer cell lines CaSki and HeLa were obtained from the American Type Culture Collection (ATCC, Manassas, VA, USA). SP2/0-Ag14 mouse myeloma cells were obtained from Shanghai Binsuirtez Biotechnology Co., Ltd. All cells were cultured in Dulbecco’s Modified Eagle Medium (DMEM, Invitrogen, Waltham, MA, USA) plus 10% fetal bovine serum (FBS, Biological Industries, Kibbutz Beit-Haemek, Israel). Primers were synthesized by Sangon Biotech (Shanghai, China). Polyethylene Glycol 1500 (14292800, Roche, Basel, Switzerland), Freund’s Adjuvant, Complete (F881-6, SIGMA), Freund’s Adjuvant, Incomplete (F5506-10), Hypoxanthine-Aminopterin-Thymidine (HAT) MediaSupplement (50×) Hybri-MaxTM (H0262-10, SIGMA), Hypoxanthine and Thymidine (HT) MediaSupplement (50×) Hybri-MaxTM (H137-10, SIGMA), goat anti-mouse IgG-horseradish peroxidase (HRP) (Invitrogen), tetramethylbenzidine (TMB) chromogen solution for ELISA (PR1210, Solarbio, Beijing, China), a Peroxidase Labeling Kit-NH_2_ (LK51, Dojindo, Shanghai, China), a Biotin Labeling Kit-NH_2_ (LK55, Dojindo), and a Smart Electrochemiluminescence (ECL) Basic Luminol (Smart-Lifesciences, Changzhou, China) were obtained. Normal cervical tissues, HPV16/18-positive cervical cancer tissues, and paraffin sections were provided by The Ninth People’s Hospital of Chongqing. One paraffin tissue section from a patient with HPV16-positive cervical squamous cell invasive carcinoma in stage Ib and one paraffin tissue section from a patient with HPV18-positive cervical squamous adenocarcinoma in stage IIa were used in the immunohistochemistry (IHC) assay. Clinical specimens used in chemiluminescence immunoassay included 4 cases of normal cervical tissue masses derived from 4 patients with uterine fibroids, 4 cases of cervical cancer tissues derived from 4 patients with stage Ib HPV52-positive cervical squamous cell invasive carcinoma, 7 cases of cervical cancer tissues derived from patients with stage Ib HPV16-positive cervical squamous cell carcinoma, and 5 cases of cervical cancer tissues derived from stage IIa HPV16-positive cervical cancers. All cancerous specimens were obtained by using the extensive hysterectomy, while normal specimens were obtained by using the panhysterectomy. A Mini Shaker MH-1 microplate oscillator (Kylin-Bell Lab Instruments, Haimen, Jiangsu, China), a 1510 MULTISKAN GO full-wavelength microplate reader (Thermo Fisher Scientific, Waltham, MA, USA), and an Infinite^®^ M200 PRO microplate reader (TECAN, Männedorf, Switzerland) were used for general experiments.

### 2.2. Preparation of HPV16 E7-HIS Fusion Oncoprotein

According to the full-length *HPV16 E7* gene, specific primers with 6 histidine (HIS) tags were designed. The sequences of primers F and R were as follows: F: 5′-GCCCATGGACCATCACCATCACCATATGCATGGAGATACACCTAC-3′; R: 5′-GCAAGCTTTTATGGTTTCTGYGAACAGATGGGGCACAC-3′. Using the total genome DNA of CaSki cells, which was pre-extracted by using the Tissue/Cell DNA Extraction Kit (Shanghai Huashun Biothech Co. ltd., China), as a template, the full-length *HPV16 E7* gene was amplified by PCR. The gradient PCR reaction conditions were performed as follows: 94 °C pre-denaturation for 5 min; 94 °C for 30 s; 10 annealing temperatures, 55.1, 55.5, 56.3, 57.7, 59.4, 61.4, 63.3, 65.3, 67.6, 69.0, 69.7, and 70.2 °C for 30 s, respectively; 35 cycles of 72 °C for 60 s; and 72 °C extension for 7 min. The 322 bp product included the full-length *HPV16 E7* gene of 297 bp, plus 25 bp of the following bases: 2 protective bases, GC; NcoI enzyme restriction sites, CCATGG and 17 bases of histidine tag gene (ACCATCACCATCACCAT), which was without one base C after the frameshift. The amplified product was digested with enzymes and identified by sequencing, and the gene was inserted into the plasmid pET-28a(+) containing T7 promoter to construct an expression vector. Then it was transformed into competent cells Rosetta (DE3) pLysS, and the engineered bacteria pET-28a(+)-HPV16 E7-Rosetta (DE3) plysS was induced by 1 mM isopropyl-β-d-thiogalactoside (IPTG; Sangon, Shanghai, China) in Luria-Bertani (LB) medium at 37 °C for 4 h. Then the bacterial solution was centrifuged, and 12% sodium dodecyl sulfate polyacrylamide gel electrophoresis (SDS-PAGE) was used to determine the protein expression level and expression form. Nickel column affinity chromatography was used for the purification of the HPV16 E7-HIS recombinant oncoprotein. The secondary structure of the purified HPV16 E7-HIS fusion oncoprotein was determined by circular dichroism chromatography.

### 2.3. Preparation of Mouse anti-HPV16 E7-HIS Fusion Oncoprotein mAbs

The HPV16 E7-HIS fusion oncoprotein was injected at a dose of 100 μg per BALB/c mouse through the footpad of the hind paw and the abdominal cavity. Footpad immunization was used to stimulate B lymphocyte proliferation and antibody production in the inguinal lymph nodes of the mice. Abdominal immunization was used to mainly stimulate mouse B lymphocyte proliferation and antibody production in the spleen. Inguinal lymph node cell suspension and spleen cell suspension of the same mouse, both of which are with high serum antibody titers, were respectively performed to fuse with SP2/0-Ag14 cells. The animals were raised in a specific pathogen free (SPF) room and the feeding conditions were strictly standardized. Before the antibodies were collected and the mice sacrificed, nasal anesthesia (isoflurane) was used to reduce their pain. The animal experiment was pre-approved by the Systems Biology and Institutional Animal Care and Use Committees of Southwest University (IACUC No.: 20180505-07; Approval date: 5 May 2018) and supervised by the Institutional Animal Care and Use Committees of the Institute of Sericulture. Monoclonal antibody subtypes were identified using mouse monoclonal antibody isotyping reagents (SIGMA). Ammonium sulfate precipitation, Protein G, Protein A, and IgM affinity chromatography, and gel chromatography were used to purify the mAb ascites, thereby obtaining several mouse mAbs for the HPV16 E7 oncoprotein with high purity.

### 2.4. Characterization of Mouse anti-HPV16 E7-HIS Fusion Oncoprotein mAbs by Using Immunocytochemistry (ICC), Immunofluorescence (IF), Immunohistochemistry (IHC), and Western Blot (WB)

CaSki and HeLa cell lines were cultured on the Fisherbrand microscope cover glasses (Cat. No. 125-45-83; Thermo Fisher) in 24-well plates with 20,000 cells per well. The mAbs were performed in the ICC and IF reactions. The SP2/0-Ag14 cell supernatant was used as a negative control. Additionally, a control without a primary antibody as a blank control was also set.

The detailed steps of the immunocytochemical reaction were as follows: CaSki and Hela cells cultured on the cover glasses in 24-well plates were fixed with 4% paraformaldehyde for 15 min at room temperature (RT). After washing, 3% H_2_O_2_ was used to eliminate endogenous peroxidase activity at RT for 20 min. Then 200 µL 1.95, 6.25, 50 µg/mL 79A11, and 69E2 primary antibodies were added to the corresponding wells, respectively. Fifty microliters of magnifying agent (Reagent I in the Dolink-2 Plus ^®^ Polymer HRP Detection System from Zsbio Inc., Beijing, China, Cat. No. PV-9002) was added to each well, and incubated at 37 °C for 1 h. One hundred and fifty microliters of secondary antibody HRP-labeled anti-mouse IgG polymer (Reagent II in the Dolink-2 Plus ^®^ Polymer HRP Detection System) with phosphate buffer saline (PBS) at 1:15,000 was added and incubated at 37 °C for 1 h. A diaminobenzadine (DAB; Cat. No.: ZLI-9018, ZsBio) color development solution was used for coloration. One hundred microliters of hematoxylin staining was used for 6–7 min for counterstaining. After being washed with water for 3 times, 100 µL 1% hydrochloric acid alcohol was used for differentiation. Then 0.2% ammonium hydroxide was used for bluing before washing with water under a microscope.

Preliminary application of mAbs 79A11 and 69E2 in indirect IF was performed as follows: CaSki and HeLa cells were cultured on the cover glasses. Ten micrograms/milliliter of primary antibodies (79A11 and 69E2) with PBS were added, and placed at 4 °C overnight for 18–20 h. Then the cells were incubated at RT for a while and then at 37 °C for 1.5 h. After washing with PBS containing 0.05% Tween 20 (PBST) 5 times, each time for 3 min, and 100 μL PBS-diluted fluorescein-labeled goat anti-mouse IgG at 1:1000, the sample was incubated at RT for 2 h. Then it was washed 5 times with PBST for 3 min each time. Afterwards, 100 µL 5 μg/ml 4′,6-diamidino-2-phenylindole (DAPI) staining solution (Beyotime, Shanghai, China) was added and the sample incubated at RT for 20–30 min, and then washed 5 times with PBST for 3 min each time. Then the cells were observed under a fluorescence microscope.

HPV16-positive cervical squamous cancer and HPV18-positive cervical cancer adenocarcinoma paraffin sections were used in IHC. Detailed protocols were as follows: paraffin specimen sections were heated at 60 °C for 2 h. Multiple steps were performed for dewaxing and hydration: xylene I for 15 min, xylene II for 15 min, 1/2 xylene (xylene:absolute ethanol = 1:1) for 10 min, absolute ethanol for 10 min, and 95% ethanol 5 min, 85% ethanol for 5 min, 75% ethanol for 5 min, water for 5 min, and PBS washing 3 times for 5 min each time. An amount of 0.01 M PBS containing 0.5% Triton X-100 (pH = 7.2) was incubated for 20 min at RT and then washed 3 times in PBS for 5 min each time. A sodium citrate repair solution was used to repair antigens in a water bath at 85 °C for 45 min. Three percent H_2_O_2_ was used to eliminate endogenous peroxidase activity at RT for 20 min. An amount of 0.01 M PBS containing 5% goat serum (pH = 7.2) was incubated for 60 min at RT. Then 200 μL of 2 mAbs 79A11 and 69E2 at 8.95 μg/mL and 50 μg/mL, respectively, was added. The cover glasses were incubated overnight at 4 °C. Then 200 μL reagent 1 (polymer adjuvant) in the Dolink-2 Plus ^®^ Polymer HRP Detection System was added and incubated at RT for 20 min and washed 3 times for 5 min each time. Then 200 μL reagent 2 (horseradish-labeled anti-mouse IgG polymer) was added and a DAB color development kit was used for coloration before the samples were observed under a microscope.

Western Blot was performed by using the IgM subtype mAb 79A11 and the IgG2a subtype mAb 69E2 to react with proteins extracted from CaSki and HeLa, respectively. A histidine-tagged human c-Myc (HIS-c-Myc) was used as a control to detect whether the mAbs 79A11 and 69E2 reacted with the HIS-tag. Detailed information is shown as follows: CaSki and HeLa cells were cultured and collected, and the total protein in CaSki and HeLa cells was extracted by using the Radioimmunoprecipitation Assay (RIPA) Lysis Buffer (Beyotime). Then the samples were separated by 12% SDS-PAGE, and the blots were transferred from the gel to nitrocellulose (NC) membrane. The NC membrane was blocked with 5% skim milk, and the primary antibodies 79A11 or 69E2 at 10 ug/mL were used to react with the protein on the NC membrane. Then the secondary antibody goat anti-mouse IgG-HRP was added, and photographed after exposure.

### 2.5. Characterization of Mouse anti-HPV16 E7-HIS Fusion Oncoprotein mAbs

The IgM subtype mAb 79A11 and IgG2a subtype 69E2 hybridoma cells were sent to Genscript (Nanjing, China) to determine the base sequence and predicted amino acid sequence. Briefly, total RNA was isolated from the hybridoma cells following the technical manual of TRIzol^®^ Reagent (Ambion, Cat. No. 15596-026). Total RNA was then reverse-transcribed into cDNA using either isotype-specific anti-sense primers or universal primers following the technical manual of the PrimeScript^TM^ 1st Strand cDNA Synthesis Kit (Takara, Cat. No. 6110A). Antibody fragments of VH, VL, CH, and CL were amplified according to the GenScript standard operating procedure (SOP) for rapid amplification of cDNA ends (RACE). Amplified antibody fragments were cloned into a standard cloning vector separately. Colony PCR was performed to screen for clones with inserts of correct sizes. No less than 5 colonies with inserts of correct sizes were sequenced for each fragment. The sequences of the different clones were aligned and the consensus sequences were provided.

Affinity measurement was performed by GenScript Inc. (Nanjing, China). Briefly, the immobilization of HPV16 E7 was performed under 25 °C while 4-(2-hydroxyethyl)-1-piperazineethanesulfonic acid (HEPES) buffered saline with ethylene diamine tetraacetic acid (EDTA) and Polysorbate 20 (HBS-EP) buffer (10 mM HEPES, 150 mM NaCl, 3 mM EDTA and 0.005% Tween-20) was used as the running buffer. The sensor chip surface of flow cells 1 and 2 were activated by freshly mixed 50 mM N-hydroxysuccinimide (NHS) and 200 mM 1-ethyl-3-(3-dimethylaminopropyl) carbodiimide hydrochloride (EDC) for 420 s (10 µL/min). Afterwards, HPV16 E7 diluted in 10 mM NaAC (pH 4.5) was injected into flow cell 2 to achieve conjugation of the maximum (MAX) response unit respectively, while flow cell 1 was set as blank. After the amine coupling reaction, the remaining active coupling sites on the chip surface were blocked with a 420 s injection of 1 M ethanolamine hydrochloride. The assay was performed at 25 °C and the running buffer was HBS-EP: 10 mM HEPES, 150 mM NaCl, 3 mM EDTA, and 0.05% Tween 20, pH 7.4 (GE Healthcare). Diluted 69E2 (IgG2a) and 79A11 (IgM) was injected over the surface as an association phase, followed by injecting running buffer as a dissociation phase. All the data were processed using the Biacore 8K evaluation software version 1.1 (GE Healthcare). Flow cell 1 and the blank injection of buffer in each cycle were used as double reference for response units (RUs) subtraction.

Dominant linear B-cell epitopes of mAbs was performed by indirect ELISA with 15 overlapping peptides that were 18 amino acids (aa) long with overlapping stretches of 6 aa. The HPV16 E7 protein sequence in the CaSki cell line on The National Center for Biotechnology Information (NCBI) website (ID: NC-001526.2) was used to design the overlapping peptides, which was subsequently synthesized by ChinaPeptides (Shanghai, China) with a purity greater than 95%. The synthesized overlapping peptides were dissolved in dimethyl sulfoxide (DMSO) to 1 mg/mL storage concentration and stored at −80 °C. The steps for indirect ELISA are shown as below: (1) Coating antigen: the bovine serum albumin (BSA) was set as a negative control protein, and a pool of 15 peptides mixed at a ratio of 1:1 was set as a positive control protein. The coating contents of 15 overlapping peptides, the negative control protein, and the positive control protein were all added as 5 μg per well. After incubating overnight at 4 °C, the plate was washed 4 times with PBST for 3 min each time; (2) Blocking: 250 μL 1.5% BSA in PBST was added and incubated at 37 °C for 2 h, and the plate was washed 3 times with PBST for 3 min each time; (3) Primary antibody incubation: the concentration of mAb 79A11 was 1.65 mg/mL, diluted 1:8000; the concentration of mAb 69E2 was 1.84 mg/mL, diluted 1:8000; 100 μL/well, 37 ° C for 1 h, washed with PBST for 4 times, 3 min for each time; (4) Secondary antibody incubation: anti-mouse IgG and HRP-linked antibody (Cat. No. 7076S, CST, Danvers, MA, USA) was diluted at 1:3000, 100 μL/well, 37 °C for 45 min, and then washed with PBST for 4 times, 3 min for each time; (5) TMB coloration: A and B solutions in the TMB two-component color development kit (Cat. No. PR1210, Solibao, Beijing, China) were diluted at 1:1, 100 μL/well, 37 °C for 5–8 min. Then 2 M H_2_SO_4_, 50 μL/well was added to stop the reaction and the optical density at 450 nm (OD_450nm_) value was detected.

Dominant linear B-cell epitopes of mAbs were screened by indirect competition ELISA, which consisted of 3 experiments: a criss-cross serial dilution analysis of indirect ELISA and 2 indirect competition ELISA experiments with overlapping peptides at 1 μM and 10 μM, respectively. Firstly, the coating concentration of antigen HPV16E7 fusion protien and response concentration of mAbs 79A11 and 69E2 were selected by using a criss-cross serial dilution analysis of indirect ELISA checkerboard experiment at an OD_450nm_ value between 0.7 and 0.8. Then antigen HPV16E7 fusion protein were coated at optimized concentration, and double optimal concentrations of the mAbs were mixed with 15 overlapping peptides or BSA of 2 μM at a ratio of 1:1. After reacting at 25 °C for 30 min, the mixture was used to react with the antigen HPV16 E7 fusion protein, and overlapping peptides inhibited by the mAbs 79A11 and 69E2 were selected. The inhibition rate (IR) was used to determine the results of indirect competition ELISA. IR = ((OD_450nm_ value of BSA − OD_450nm_ value of overlapping peptide samples) × 100%)/(OD_450nm_ value of BSA). IR ≥ 50% was considered positive. When the overlapping peptide was 1 μM, both mAbs 79A11 and 69E2 could inhibit part of the overlapping peptide, and the inhibition rates of most of the inhibited overlapping peptides were less than 50%, which is negative. Therefore, the concentration of overlapping peptides needed to be increased to 10 μM. The methods and steps of overlapping peptides at 10 μM were as follows: (1) Coating the antigen HPV16 E7 fusion protein: for mAb 79A11, when the coating concentration of the antigen HPV16 E7 protein was 1 μg/mL, the corresponding concentration of 79A11 mAb was 1 μg/mL; for the mAb 69E2, when the coating concentration of the antigen HPV16 E7 was 0.25 μg/mL, the corresponding concentration of the mAb 69E2 was 0.125 μg/mL. The plates were coated with 100 μL HPV16 E7 oncoprotein antigen at 4 °C overnight, and then washed with PBST 4 times, for 3 min each time; (2) Blocking: 1.0% BSA with PBST was prepared and incubated at 37 °C for 2 h, and then the plates were washed 3 times with PBST for 3 min each time; (3) Incubation with a mixture of primary antibody and overlapping peptides: mAbs 79A11 and 69E2 at 2 μg/mL and 0.25 μg/mL respectively were prepared, and several overlapping peptides inhibited by mAbs were selected, while 1–2 overlapping peptides that were not inhibited by the mAbs and BSA were used as negative controls. Afterwards, 2 μg/mL of mAb 79A11 and 0.25 μg/mL of mAb 69E2 were respectively mixed with 20 μM overlapping peptide, 20 mg/mL BSA, and PBST at 1:1, and incubated at 25 °C by shaking for 30 min, then these mixtures were added to the microplates coated with the HPV16 E7 fusion protein, 100 μL/well, and incubated at 37 °C for 1 h. Then the plates were washed 4 times with PBST for 3 min each time; (4) Secondary antibody incubation and TMB coloration were performed as indirect ELISA described above.

Using the HPV45 E7 dimer as a model in SWISS-MODEL homology modeling, a structural structure model of amino acids 46–97 of HPV16 E7 was obtained. Then the PyMOL (The PyMOL Molecular Graphics System 2.2.0, New York, NY, USA) was used to present the three-dimensional (3D) structural model of HPV16 E7. The software Molecular Evolutionary Genetics Analysis 7.0 (MEGA 7.0) was used to compare the amino acid sequences of the 3 exposed peptides of the HPV16 E7 protein with that of the other 30 HPV16 strains downloaded from the NCBI website.

### 2.6. Monoclonal Antibody Pairing

A double-antibody sandwich ELISA assay was used for pairing experiments. Unlabeled mAbs were used as capture antibodies, HRP-labeled mAbs as detection antibodies, and TMB substrate was used for coloration. The OD_450 nm_ value was used to indirectly measure the antigen content. HRP was used to label 8 high effective mouse anti-HPV16 E7-HIS oncoprotein mAbs according to the instructions of the Peroxidase Labeling Kit-NH_2_ (Dojindo, Sahnghai, China). HRP-labeled mAbs were used as detection antibodies, and mAb pairing experiments were performed with unlabeled mAbs as capture antibodies. A total of 56 pairs were paired. The pair of mAbs with the strongest pairing signal was subjected to conventional double-antibody sandwich ELISA to quantitatively detect the lowest limitation of the HPV16 E7-HIS oncoprotein.

### 2.7. Establishment of a Double-Antibody Sandwich ELISA Method Based on HRP-Labeled mAb and TMB Detection System for Quantitative Detection of HPV16 E7-HIS Fusion Oncogenic Protein

Criss-cross serial dilution analysis was performed to obtain the optimal working concentration of the capture antibody, the optimized antigen concentration, and reaction time between the capture antibody and the antigen, thereby establishing a reference curve under the optimized conditions to obtain a linear regression equation. Briefly, a specified concentration of the 79A11 capture antibody was coated and incubated at 37 °C for 2 h, then transferred to 4 °C for 12–16 h, and then washed 3 times with PBST, each time for 3–5 min. Then 250 μL 5% skim milk dissolved in PBST was incubated at 37 °C for the indicated time for blocking, and the plate was washed 3 times with PBST for 3–5 min each time. One hundred milliliters of indicated concentrations of HPV16 E7 antigen were incubated for 2 h then washed 3 times with PBST for 3–5 min each time. One hundred milliliters of 1 μg/mL HRP-69E2 in PBST were incubated at 37 °C for 1 h, and washed 3 times with PBST, each time for 3–5 min. Then TMB was used for coloration and 2 M H_2_SO_4_ was used to stop the reaction. The OD_450nm_ value was detected by a microplate reader.

### 2.8. Establishment of the LSAB-ELISA and Luminol Detection System for Quantitative Detection of HPV16 E7-HIS Fusion Oncogenic Protein

According to the LK55 Biotin Labeling Kit-NH_2_ instructions, biotin was used to label the antibody 69E2 to obtain biotinylated detection antibody Biotin-69E2. Criss-cross serial dilution analysis by using a double-antibody sandwich ELISA was performed to optimize the optimal working concentrations of the capture mAb 79A11 and detection of the antibody Biotin-69E2, blocking conditions, and reaction time of the antigen HPV16 E7-HIS fusion oncoprotein with the antibody 79A11 (subtype IgM). The coating concentrations of the capture antibody 79A11 were 0.5, 1, 2, and 4 μg/mL, respectively. The concentrations of Biotin-69E2 were 0.25, 0.5, and 1 μg/mL, respectively. The blocking agents were 0.25% BSA, 1% BSA, and 5% skim milk, respectively. Blocking times were 30, 60, and 120 min, respectively. The reaction times of the antigen and capture antibody were 0.5, 1.5, and 2 h. Nine dilution points (0, 25, 50, 100, 200, 400, 600, 800, and 1000 ng per well) of the antigen were used to obtain the reference curve and determination coefficient. The upper detection limitation was determined by using 8 dilution points (0, 0.5, 1, 2, 4, 6, 8, and 10 μg of the antigen per well). The Chemiluminescence (relative light unit, RLU) of each reaction was detected by a microplate reader.

### 2.9. HPV16 E7 Oncoprotein Determination by Using the LSAB-ELISA and Luminol Detection System

The total protein of normal cervical tissue specimens, HPV16 positive cervical cancer specimens, and paracancerous specimens were extracted by using the RIPA Lysis Buffer. The Bicinchoninic Acid (BCA) Protein Assay Kit (Beyotime) was used to determine the total protein concentration. A reference curve was prepared under the optimal conditions and the HPV16 E7 oncogenic protein in 20 μg total protein of cervical cancer specimens was quantitatively detected by the established LSAB-ELISA and luminol detection system.

## 3. Results

### 3.1. Preparation of HPV16 E7-HIS Fusion Oncoprotein

**Figure 1:**
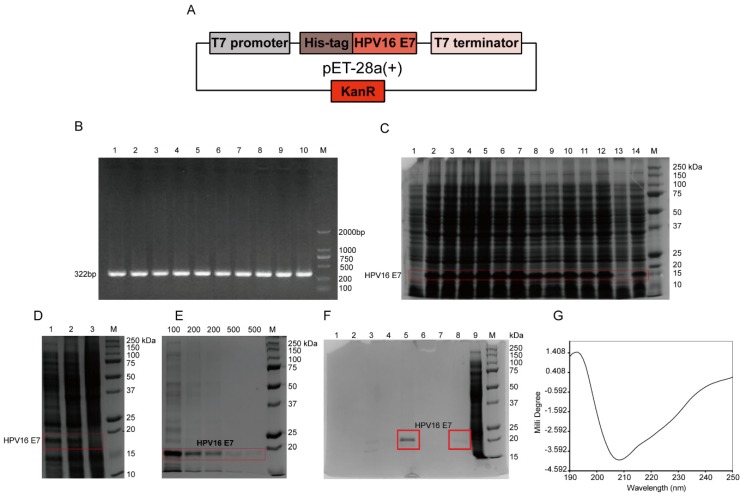
Preparation of the HPV16 E7-HIS fusion oncoprotein. (**A**) *HPV16 E7* gene with T7 promoter, HIS-tag, and kanamycin resistance was recombined into plasmid pET-28a(+) to construct pET-28a(+)-HPV16 E7 recombinant plasmid. Different colors represent different protein expressing elements in the vector. (**B**) Amplification of the *HPV16 E7* gene by gradient PCR. Lanes 1–10 were the 322bp PCR product of the *HPV16 E7* gene at an annealing temperature of 50–70 °C, and M was the DNA Marker DL-2000. (**C**) 15% SDS-PAGE of the HPV16 E7 fusion oncoprotein-induced expression in prokaryotic cells. M was the protein marker; lanes 1 and 13 were empty carriers; lanes 2–5, 6–9, 10–12, and 14 were samples performed at 16 °C, 30 °C, and 37 °C, respectively, induced by 0.1, 0.5, 0.75, and 1.0 mM IPTG, respectively. The red box represents the bands of HPV16 E7. (**D**) 12% SDS-PAGE of expression form of the HPV16 E7 fusion protein. Lanes 1, 2, 3, and M were bactericidal solution, supernatant, precipitation, and protein marker, respectively. The red box represents the bands of HPV16 E7. (**E**) 12% SDS-PAGE of the HPV16 E7 fusion protein purified by nickel column. Lanes 100, 200, 500, and M were samples performed by using100 mM, 200 mM, and 500 mM imidazole eluate, and protein marker, respectively. The red box represents the bands of HPV16 E7. (**F**) 12% SDS-PAGE of the HPV16 E7-HIS fusion oncoprotein purified by DEAE column. Lanes 1 to 5 were the 200 mM imidazole-eluted peak sample of the HPV16 E7 protein purified by DEAE column penetration, re-equilibrium, 0.3 M NaCl elution at peak tip, 0.3 M NaCl elution at falling peak, and 0.5 M NaCl elution at peak tip, respectively; lanes 6 to 8 were 500 mM imidazole-eluted peak samples of the HPV16 E7 protein purified by DEAE column penetration, re-equilibrium, and 0.5 M NaCl elution peak tip, respectively; lane 9 was bacterial lysate of the IPTG-induced engineered bacteria pET-28a(+)-HPV16E7-Rosetta (DE3) pLysS; M was protein marker. The red box represents the bands of HPV16 E7. (**G**) The purified HPV16 E7-HIS fusion proteins were determined by circular dichromatography. HPV, human papillomavirus; HIS, histidine; IPTG, isopropyl-β-d-thiogalactoside.

The *HPV16 E7* gene with HIS-tag was inserted into a pET-28a(+) plasmid to construct a recombinant plasmid pET-28a(+)-HPV16 E7 (Figure 1A). Nucleic acid electrophoresis found that only a band with a molecular weight (MW) of 322 bp was amplified (Figure 1B). The predicted amino acid (aa) sequence of the HPV16 E7-HIS fusion oncoprotein expressed by the recombinant *HPV16 E7* gene was MDHHHHHMHGDTPTLHEYMLDLQPETTDLYCYEQFNDSSEEEDEIDGPAGQAEPDRAHYNIVTFCCKCDSTLRLCVQSTHVDIRTLEDLLMGTLGIVCPICSQKP, which contains a total of 105 aa, including the first 7 amino acids (methionine expressed by one ATG expressed by Nco I, aspartic acid expressed by frameshift GAC, and 5 histidine tags) and 98 aa that constitute the HPV16 E7-HIS fusion oncoprotein. The predicted isoelectric point (PI) of the HPV16 E7-HIS fusion oncoprotein monomer was 4.59 and the predicted MW was 11988.32 Da. The DNA sequence identification and comparison (Figures S1 and S2 in Supplementary Materials S2) with the software MEGA7.0 revealed that: (1) The HPV16 E7 gene sequence of the recombinant plasmid pET-28a(+)-HPV16 E7 differed from the full-length *HPV16 E7* gene in the HPV16-type CaSki cell line by 2 bases, while the amino acid sequences were different by 1 amino acid (28F vs. 28L); (2) The *HPV16 E7* gene sequence of the recombinant plasmid pET-28a(+)-HPV16 E7 differed from the full-length *HPV16 E7* gene in the HPV16-type SiHa cell line by 1 base, but the amino acid sequences were consistent.

Then the engineered bacteria pET-28a(+)-HPV16 E7-Rosetta (DE3) pLysS and the empty vector pET-28a(+)-Rosetta (DE3) pLysS were induced and expressed, and were analyzed by 15% SDS-PAGE. The results showed that a higher content band appeared at about 15 kDa after 0.1, 0.5, 0.75, and 1.0 mM IPTG induction at 16, 30, and 37 °C, compared with the empty vector. The predicted MW of the HPV16 E7-HIS fusion oncoprotein was about 12 kDa; however, we found a 15 kDa band that has a molecular weight close to the natural HPV16 E7 protein, which was consistent with previous reports that the MW of HPV16 E7 migrated from 14 to 21 kDa in SDS-PAGE due to the protein negative charge [14] (Figure 1C), indicating the success of the expression of the HPV16 E7-HIS fusion oncoprotein. Additionally, the expressed HPV16 E7-HIS fusion oncoprotein appeared in the supernatant, indicating that the protein is soluble (Figure 1D) and may have activity.

SDS-PAGE showed that there was a band of HPV16 E7-HIS fusion oncoprotein with some unspecific bands after purification with 100 mM imidazole elution by using a nickel column affinity layer. With the increasing concentrations of imidazole, there were still some nonspecific bands (Figure 1E). To obtain a purer HPV16 E7-HIS fusion oncoprotein, the samples were subsequently purified by diethylaminoethyl (DEAE) anion exchange chromatography. Twelve percent SDS-PAGE revealed that a band appeared at about 18 kDa after elution with 0.5 M NaCl (Figure 1F), indicating that a high-purity HPV16 E7-HIS fusion protein was obtained. The circular dichromatography determined that the HPV16 E7-HIS fusion oncoprotein had an alpha helix structure in the secondary structure (Figure 1G). These results show that we successfully obtained a qualified HPV16 E7-HIS fusion oncoprotein.

### 3.2. Preparation of Anti-HPV16 E7 Protein mAbs

**Figure 2:**
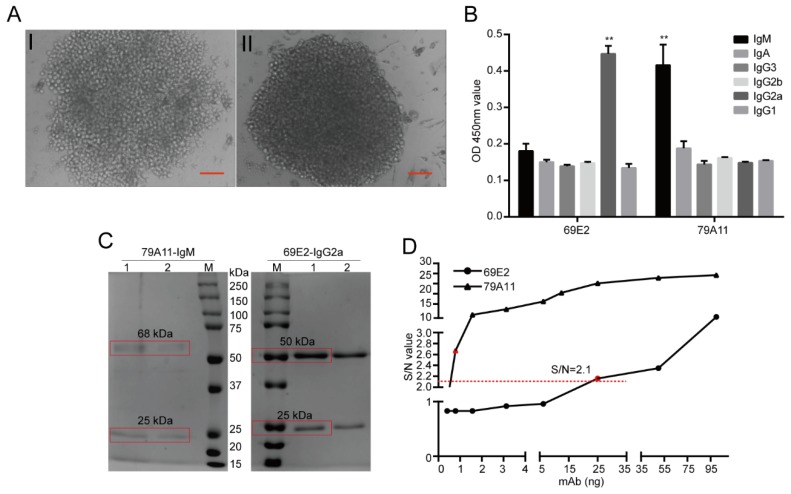
Preparation of the mAbs against the HPV16 E7-HIS fusion oncoprotein. (**A**) I and II were hybridoma cells at the 8th day after SP2/ 0-Ag14 were fused with immuned BABL/c lymph node cells and spleen cells, respectively. Scale bar=500 μM (**B**) Subtypes were identified as IgM and IgG2a subtypes by using the mouse monoclonal antibody isotyping reagents (SIGMA). ** represents *p* < 0,01, compared with other kinds of immunoglobulins (Igs) by using the One-way Analysis of Variance (ANOVA) with post-hoc Tukey Honestly Significant Difference (HSD) for multiple comparison. (**C**) 12% SDS-PAGE of the IgM subtype mAb 79A11 purified by Protein A. 25 and 68 kDa were the light and heavy chains of IgM mAbs (left); 12% SDS-PAGE of the IgG2a subtype mAb 69E2 purified by Protein G. 25 kDa and 50 kDa were the light and heavy chains of the IgG2a subtype mAbs (right). The red box represents the bands of HPV16 E7. (**D**) Indirect ELISA assay for the purified mAb titer. mAb, Monoclonal antibody. The red dotted line presents S/N ratio = 2.1.

The lymph node cells and spleen cells of mice immunized with the HPV16E7-HIS fusion protein were fused with SP2/0-Ag14 cells by the hybridoma technique, and more than 20 monoclonal hybridoma cell lines were obtained. The cell colonies were large and the growth was good (Figure 2A,B). IgM and IgG2a subtype antibodies were found by using the capture method provided by the manufacture’s protocol of an mAb Subtype Identification Kit (Sigma, Figure 2B). The IgM subtype mAb (79A11) had two bands of 25kDa and 68kDa in 10% SDS-PAGE (Figure 2C), which were consistent with the MWs of the light and heavy chains of IgM in mice, respectively. The IgG2a subtype mAb (69E2) had two bands of 25 kDa and 50 kDa (Figure 2C right), which were consistent with the MWs of the light and heavy chains of mouse IgG2a, indicating that the purity of the purified 79A11 and 69E2 mAbs were high. The antibody titers of the purified 79A11 and 69E2 mAbs were 6.25 ng and 0.78 ng, respectively, which was determined by indirect ELISA with positive signals with a signal-to-noise (S/N) ratio ≥ 2.1 (Figure 2D).

### 3.3. Characterization of the Anti-HPV16 E7 Protein mAbs

**Figure 3:**
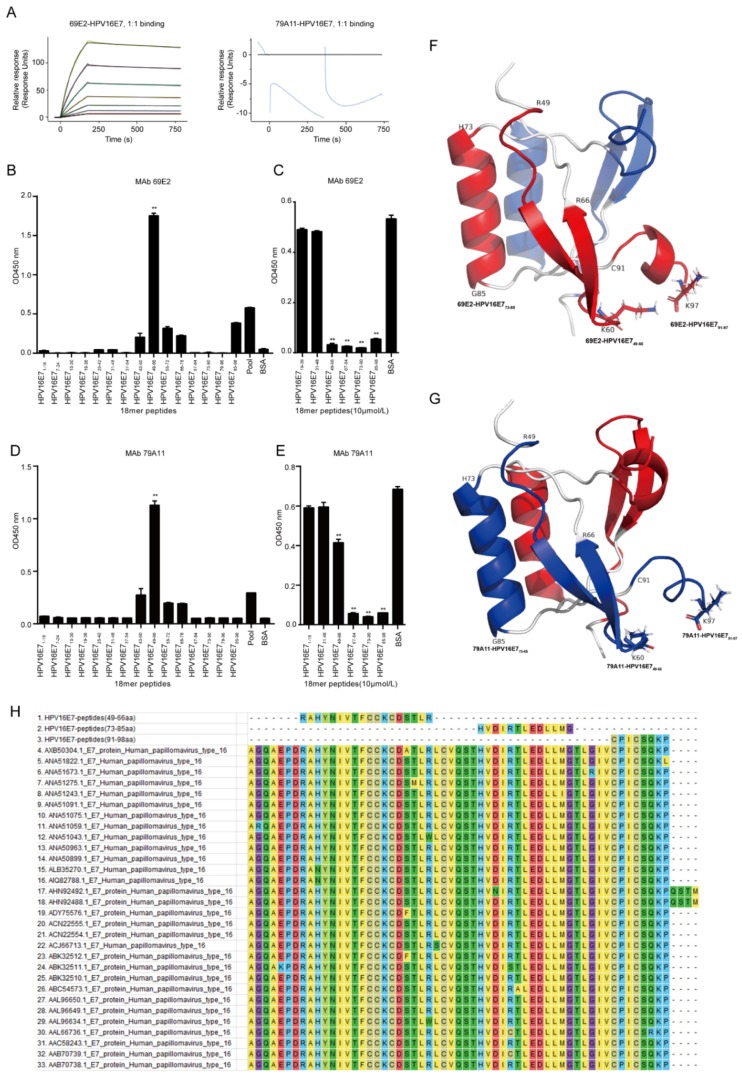
Characterization of the anti-HPV16 E7-HIS fusion oncoprotein mAbs. (**A**) Affinity measurement of the 69E2 and 79A11 mAbs. Running configuration: immobilization; ligand; HPV16 E7; immobilization level; 1972 Ru. Association and dissociation for 69E2: association contact time, 180 s; dissociation contact time, 600 s; flow rate, 30 μL/min; sample concentrations, 7.8125, 15.625, 31.25, 62.5, 125, 250, and 500 nM. Association and dissociation for 69E2: Association contact time, 180 s; dissociation contact time, 120 s; flow rate, 30 μL/min; sample concentrations, 500 nM. (**B**,**C**) Screening of dominant linear B-cell epitope of the anti-HPV16 E7-HIS fusion oncoprotein mAb 69E2 was used by indirect ELISA with overlapping peptides. ** represents *p* < 0,01. Significance was performed by using the One-way ANOVA with post-hoc Tukey HSD for multiple comparison. (**D**,**E**) Screening of dominant linear B-cell epitopes of mAb 79A11 was used by indirect competition ELISA. ** represents *p* < 0.01. Significance was performed by using the One-way ANOVA with post-hoc Tukey HSD for multiple comparison. (**F**,**G**) Location of specific epitopes of mAbs 69E2 and 79A11 on the 3D crystal structure modeling map of the HPV16 E7 protein. The three specific binding peptides HPV16 E7_49–66_, HPV16 E7_73–85_, and HPV16 E7_91–97_ of mAb 79A11 are marked in blue, and the three specific binding peptides HPV16 E7_49–66_, HPV16 E7_73–85_, and HPV16 E7_91–97_ of mAb 69E2 are marked in red. (**H**) Conservative analysis of the specific epitopes of mAbs 79A11 and 69E2 by using the software MEGA7.0.

Then the 69E2 and 79A11 hybridoma cells were sent to GenScript (Nanjing) for antibody full-length sequencing. The results (Supplementary Materials S1) showed the antibody isotype of 69E2 was mouse IgG2a, kappa, while 79A11 was IgM, kappa, which was consistent with our previous identification (Figure 2B). IgBLAST showed that these were novel antibodies that were significantly different from all the current HPV16 E7 antibodies (Figures S3–S6 in Supplementary Materials S2). Then the affinity measurement revealed that the affinity of HPV16 E7 to 69E2 was 5.60E-09 M, whereas non-specific binding was found when 79A11 (IgM) was detected for the binding of the HPV16 E7 protein (Figure 3A, Table 1). This discordance may be caused by the lysine residue in the HPV16 E7 protein, which would bind to carboxyl of the chip, blocking the binding of 79A11 to HPV16 E7.

By using the signal to noise (S/N) ratio > 2.1 as a standard of the positive result of the indirect ELISA method, the results revealed that there were 5 overlapping peptides among 15 synthetic 18 aa overlapping peptides which could react with mAb 69E2 (IgG2a). The signal of 69E2 (IgG2a) reacted with the peptide HPV16 E7_49–66_ was the strongest (*p* < 0.01), and the order of overlapping peptides with positive signals from high to low of OD_450nm_ value was HPV16 E7_49–66_ > HPV16 E7_85–98_ > HPV16 E7_55–72_ > HPV16 E7_61–78_ > HPV16 E7_43–60_ (Figure 3B). Since indirect ELISA experiments of overlapping peptides can only screen overlapping peptides of highly hydrophobic amino acids, but might omit overlapping peptides of highly hydrophilic amino acids, we used indirect competitive ELISA to screen peptides containing highly hydrophilic amino acids. The results showed that when the overlapping peptides were at 10 μM, there were 4 overlapping peptides inhibited by mAb 69E2, and the overlapping signals from strong to weak were: HPV16 E7_73–90_ > HPV16 E7_67–84_ > HPV16 E7_49–66_ > HPV16 E7_85–98_ (Figure 3C).

Similarly, there were four overlapping peptides that showed positive signals in the indirect ELISA method of overlapping peptides reacted with mAb 79A11 (IgM). The mAb 79A11 reacted with the peptide HPV16 E7_49–66_ with a strong signal. The order of positive signals of overlapping peptides from strong to weak was: HPV16 E7_49–66_ > HPV16 E7_43–60_ > HPV16 E7_55–72_ > HPV16 E7_61–78_ (Figure 3D). Indirect competitive ELISA of overlapping peptides revealed that when the overlapping peptides were at 10 μM, the 4 overlapping peptides HPV16 E7_73–90_, HPV16 E7_67–84_, HPV16 E7_85–98_, and HPV16 E7_49–66_ were significantly inhibited by mAb 79A11 (*p* < 0.01). In addition, the order of overlapping peptides were suppressed from strong to weak were: HPV16 E7_73–90_ > HPV16 E7_67–84_ > HPV16 E7_85–98_ > HPV16 E7_49–66_ (Figure 3E).

Combining the results of indirect ELISA and indirect competition ELISA with overlapping peptides, we concluded that the 69E2 and 79A11 mAbs might specifically bind to HPV16 E7 through interaction with the sequences within HPV16 E7_49–66_, HPV16 E7_67–84_, HPV16 E7_73–90_, and HPV16 E7_85–98_. However, the specific amino acids consisting of the epitopes of HPV16 E7 were not very clear.

Although the crystal structure of the HPV16 E7 protein has not been retrieved, the amino acid sequence of the HPV16 E7 protein is similar to that of the HPV45 E7 protein, with 46% sequence homology (Figure S7 in Supplementary Materials S2). The HPV45 E7 protein and the HPV16 E7 protein have similar crystal structure. The structure of HPV45 E7 protein was used as the model by using the SWISS-MODEL, and a structural crystal structure model of HPV16 E7_46–97_ was obtained. The PyMOL was used to present the 3D structure of HPV16 E7. The carboxy termini of the HPV E7 oncoprotein includes a zinc-binding domain consisting of two copies of a Cys-X-X-Cys sequence motif separated by 29 amino acid residues [15]. The E7 protein performs its function by forming dimers or multimers through the zinc-binding region at the C-terminus [16]. The two monomers of the HPV16 E7 protein were linked into a dimer with a zinc-binding region, showing a double axis of rotational symmetry. These results implied that the secondary structure of the HPV16 E7 protein was indeed an alpha helix, which was also determined by the circular dichromatography (1G).

The results of indirect ELISA and indirect competitive ELISA of overlapping peptides showed that the mAbs 79A11 and 69E2 specifically bound to four overlapping peptides: HPV16 E7_49–66_, HPV16 E7_67–84_, HPV16 E7_73–90_, and HPV16 E7_85–98_. These overlapping peptides were subsequently marked in the 3D structural modeling of the HPV16 E7 protein. The results showed that two peptides, HPV16 E7_67–72_ and HPV16 E7_86–90_, can be found in the internal of the 3D crystal structural model, while three peptides, HPV16 E7_49–66_, HPV16 E7_73–85_, and HPV16 E7_91–97_, were exposed, discontinuous, and adjacent in the same monomer (Figure 3F,G). The internal peptides cannot be an epitope, while the exposed peptides may be epitopes. The three exposed peptides were discontinuous in the same monomer, but were adjacent in space, indicating that the three specific epitopes of both mAbs 79A11 and 69E2 were conformational epitopes. Since the mAb 79A11 was an IgM-type, its affinity was not high, implying that the epitopes of mAb 79A11 were flexible epitopes. Although the mAb 69E2 was an IgG2a-type and its affinity was high, its epitopes were likely rigid epitopes.

Using the software MEGA7.0, it was found that the amino acid sequences of HPV16 E7_49–66_, HPV16 E7_73–85_, and HPV16 E7_91–97_, and three exposed specific epitopes of the two mAbs 79A11 and 69E2, were highly homologous with the amino acid sequences of the HPV16 E7 protein of 30 HPV16 strains downloaded from NCBI (Figure 3H), indicating that these exposed specific epitopes are highly conservative, and may have a promisingly high value of broad-spectrum application.

### 3.4. Effectiveness and Specificity of Anti-HPV16 E7-HIS Oncoprotein mAbs Identified by Using ICC, IF, IHC, and WB

**Figure 4:**
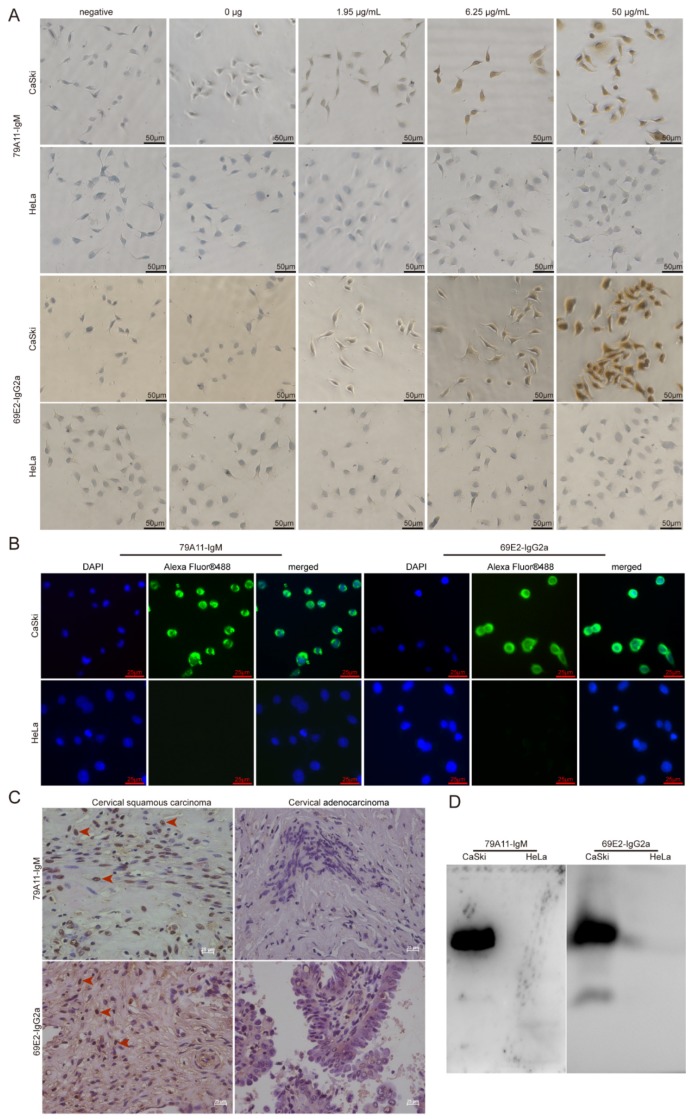
Effectiveness and specificity of the anti-HPV16 E7 protein mAbs identified by using immunocytochemistry, immunofluorescence, immunohistochemistry, and Western Blot. (**A**) The 79A11 and 69E2 antibodies at the three dosages of 1.95, 6.25, and 50 μg/mL, respectively, were used to react with the HPV16-type cervical cancer cell line CaSki and the HPV18-type cervical cancer cell line HeLa in immunocytochemical reactions. (**B**) The 79A11 and 69E2 mAbs, respectively, reacted with the CaSki and HeLa cervical cancer cell lines at a dose of 10 μg/mL in IF experiments. (**C**) The 79A11 and 69E2 mAbs reacted with the paraffin sections of HPV16 cervical squamous cell carcinoma and HPV18 cervical adenocarcinoma tissues at 8.95 and 50 μg/mL, respectively, in IHC assays. (**D**) The 79A11 and 69E2 mAbs reacted with CaSki cell protein extract at 10 μg/mL, respectively, in Western Blot experiments. IF, immunofluorescence; IHC, immunohistochemistry

The 79A11 and 69E2 mAbs were used at low, medium, and high levels (0, 1.95, 6.25, 50 μg/mL) in the ICC experiments. Typical brownish-yellow particles appeared in reaction with CaSki cells, and no brown-yellow particles in reaction with HeLa cells (Figure 4A). No brown-yellow particles appeared in negative control and blank control. It was found by IF that the mAbs, including both 79A11 and 69E2, showed a green fluorescent signal when reacted with CaSki cells at 10 μg/mL, but did not show a green fluorescent signal when they reacted with HeLa (Figure 4B). Using IHC experiments, it was found that the IgM mAb 79A11 at 8.95 μg/mL and the IgG2a mAb 69E2 at 50 μg/mL, respectively, reacted with HPV16-positive cervical cancer squamous cell carcinoma paraffin sections and showed typical positive brown particles. Brown-yellow granules did not appear in paraffin sections of HPV18 type-positive cervical cancer adenocarcinoma (Figure 4C). Western Blot experiments revealed that 10 μg/mL of IgM mAb 79A11 and IgG2a mAb 69E2 were used to react with the protein extraction solution of CaSki cells, respectively, and typical exposure bands appeared at about 18 kDa. However, no typical exposure bands appeared after these antibodies reacted with HeLa cells (Figure 4D), or HIS-c-Myc. The above data showed that the mouse anti-HPV16 E7-HIS fusion protein mAbs were effective and specific.

### 3.5. Establishment of a Double-Antibody Sandwich ELISA Based on an HRP-Labeled mAb and TMB Detection System for Quantitative Detection of HPV16 E7-HIS Fusion Oncoprotein

**Figure 5:**
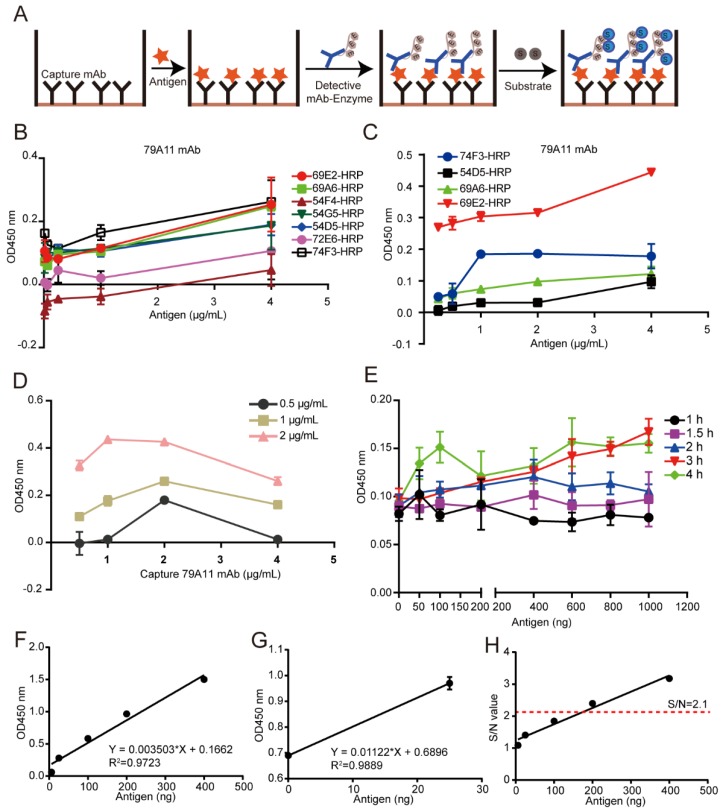
Establishment of a double-antibody sandwich ELISA based on an HRP-labeled mAb and a TMB detection system for quantitative detection of the HPV16 E7-HIS fusion oncoprotein. The red pentagram represents an antigen; the black “Y” represents a capture antibody; the blue “Y” represents a detecting antibody; E represents an HRP enzyme and S represents a substrate (TMB). (**A**) Schematic diagram of the double-antibody sandwich ELISA. The principle of a conventional double-antibody sandwich ELISA: an mAb without the labeled enzyme HRP was used as the capture antibody, and an HRP-labeled mAb was added after the antigen reaction, and the color substrate TMB was used to develop the color. The OD_450nm_ value was used to calculate the amount of antigen in the picture. (**B**) The positive result of the first mAb pairing. (**C**) The re-pairing results of four pairs of mAbs with positive signals. (**D**) The optimization of the capture antibody concentration. (**E**) The optimization of the reaction time between the antigen HPV16 E7 protein and the capture antibody. (**F**) The linear relationship diagram between the antigen HPV16 E7 protein content and the OD_450nm_ detected by double-antibody sandwich ELISA. (**G**) The regression equation for calculating the detection limit of the antigen. (**H**) The linear relationship diagram between the antigen HPV16 E7 protein content and the S/N value of antigen detected by double-antibody sandwich ELISA. HRP, horseradish peroxidase; TMC, tetramethylbenzidine.

Two mAb pairing experiments using the double-antibody sandwich ELISA method selected four pairs of mAbs with positive signals (Figure 5A), including three HRP-IgG2a-type mAbs and an IgM subtype mAb. The capture antibody was an IgM subtype mAb 79A11. The second pairing experiment found the strongest signal when 69E2 mAb reacted with 79A11 mAb (Figure 5B,C). These results indicated that 79A11 was suitable to be used as a capture antibody.

The double-antibody sandwich ELISA assay showed that the positive signals was gradually increased when the coating concentrations of the capture antibody 79A11 was increased from 1 to 2 μg/mL, but decreased at 4 μg/mL (Figure 5D), indicating that the optimal coating concentration was 2 μg/mL. Furthermore, the positive signals of the reaction between the antigen HPV16 E7-HIS fusion oncoprotein and the capture antibody were positively correlated with reaction time (Figure 5E). Taking the antigen content (ng) as the abscissa and the OD_450nm_ value as the ordinate, a reference curve was prepared. The coefficient of variation (CV) fluctuated from 0.899 to 11.7264%, and its coefficient of determination, R squared (*R*^2^) was 0.9723 (Figure 5F), indicating a good linear relationship. The equation for calculating the detection limit was Y = 0.01122 × X + 0.6896 and *R*^2^ = 0.9889, which was obtained by using no antigen and a low concentration of antigen with a CV lower than 10% as the abscissa, with the corresponding OD_450nm_ value as the ordinate (Figure 5G). The detection limit was 122.9234 ng when the S/N ratio = 3. Using the graph with the antigen as the abscissa and the S/N as the ordinate, it was also found that the corresponding antigen was greater than 100 ng when the S/N was greater than 2.1 (Figure 5H), which was consistent with the detection limit calculated in Figure 5G. These results indicated that the sensitivity of this method was not ideal, and a more sensitive detection system needs to be established.

### 3.6. Establishment of Chemiluminescence Immunoassay Based on LSAB-ELISA Method and Luminol Detection System for Quantitative Detection of HPV16 E7-HIS Fusion Oncogenic Protein

**Figure 6:**
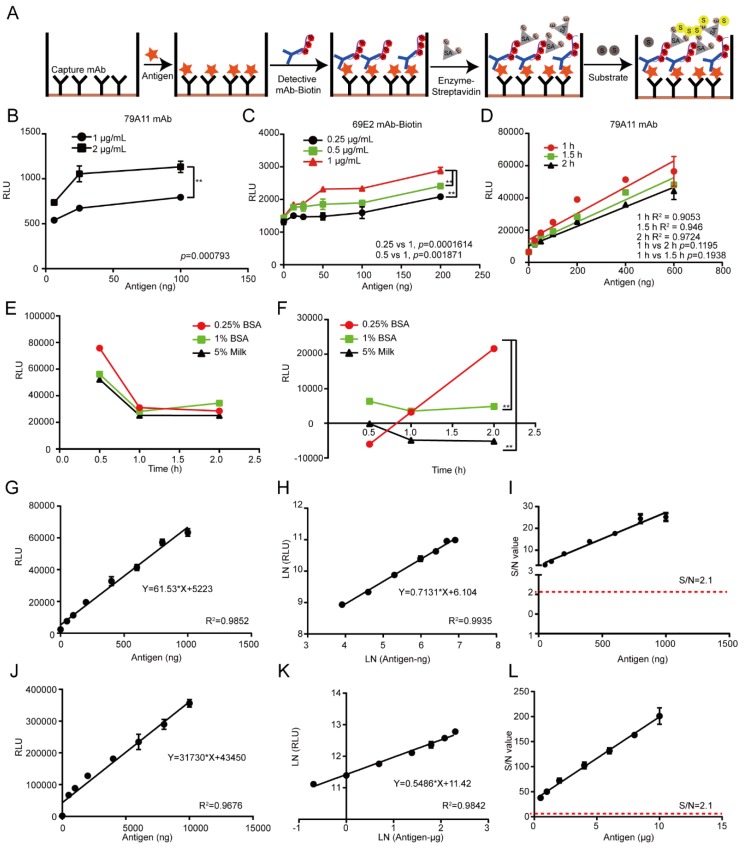
Establishment of a chemiluminescence immunoassay based on the LSAB-ELISA method and luminol detection system for quantitative detection of the HPV16 E7-HIS fusion oncogenic protein. (**A**) The principle diagram of the biotin-streptavidin double-antibody sandwich ELISA (BA-ELISA). The red pentagram represents an antigen; the black “Y” represents a capture antibody; the blue “Y” represents a detecting antibody; B represents an biotin; SA represents a streptavidin; S represents a substrate (lumonol) and E represents an HRP enzyme. (**B**) The optimal concentration of the capture antibody 79A11. (**C**) The optimized reaction concentration of the detection antibody Biotin-69E2. (**D**) The optimized reaction time between the antigen HPV16 E7 protein and the capture antibody. (**E**) The background of the three blocking agents at three time points. (**F**) Signal diagrams at three time points for the three blocking agents. (**G**–**I**) The straight-line fitting, double-log fitting, and S/N ratio of the antigen HPV16 E7-HIS oncoprotein at the ng-grade equal-difference dilution method. (**J**–**L**) The straight-line fitting, double-logarithmic fitting, and S/N ratio plots of the HPV16 E7-HIS recombinant oncoprotein at the μg-grade equal-difference dilution method. LSAB, labeled streptavidin-biotin. RLU, relative light units.

Then we tried to establish a double-antibody sandwich experiment to detect the presence of the HPV16 E7 oncoprotein (Figure 6A), and it was found that the signal of the capture antibody 79A11 (IgM subtype) at 2 μg/mL was stronger than that at 1μg/mL (Figure 6B). The signal of Biotin-69E2 gradually increased from 0.25, to 0.5, to 1 μg/mL, and the best linear relationship (*R*^2^ = 0.9724) of the antigen response time was 2 h (Figure 6C,D). The background chemiluminescence values detected by using the blocking agents (0.25% BSA, 1% BSA, and 5% skim milk) were decreased with time (Figure 6E), while its corresponding positive signals of chemiluminescence values were increased with time, and the strongest signal was blocked by 0.25% BSA for 2h (Figure 6F). When the antigen was diluted at 9 points in the ng-grade, the correlation *R*^2^ of the straight line fitting and the logarithmic fitting was 0.9852 and 0.9935, respectively (Figure 6G,H), which were all above 0.965, indicating that the linear relationship was good. The S/N ratio was > 2.1 at low antigen concentrations of 25 ng (Figure 6I), indicating that it was a reliable positive signal. When the antigen was diluted at 9 points in the μg-grade, the correlation of the straight line fit and the *R*^2^ was 0.9678 and 0.9842, both above 0.965, indicating that the linear relationship of the antigen at the μg level was good (Figure 6J,K). The S/N ratio was > 2.1 at low antigen concentrations of 0.5 μg (Figure 6L), indicating that it was a reliable positive signal. No upper detection limit was found when the antigen contents were between 1 to 10 μg at present.

### 3.7. Detection of HPV16 E7 Oncoprotein Presence in Clinical Cervical Cancer Specimens by the Established LSAB-ELISA Method and Luminol Detection System

**Figure 7:**
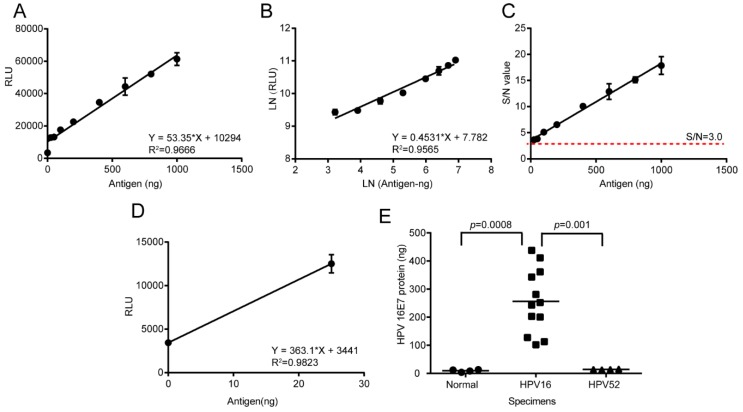
Preparation of reference curves and detection of clinical specimens by chemiluminescence immunoassay. (**A**–**C**) The straight-line fitting, double-logarithmic fitting, and S/N ratio of the reference curve for cervical cancer specimens. (**D**) The reference curve and equation for calculating the detection limit. (**E**) The HPV16 E7 content of 3 groups of tissues, including 4 normal cervical tissues, 12 HPV16-positive cervical cancer tissues and 4 HPV52-positive cervical cancer tissues. Twenty μg of total protein per specimen was used for detection. The significance was shown by using the One-way ANOVA with post-hoc Tukey HSD for multiple comparison. RLU, relative light units.

Then the effectiveness of the chemiluminescence immunoassay based on the LSAB-ELISA method and luminol detection system was tested by using clinical cancer specimens. The reference curve was prepared with 79A11 at 2 ug/mL, 0.25% BSA blocking for 2 h, the antigen ng ratio dilution with the capture antibody at 37 °C for 2 h, and Biotin-69E2 and HRP-Streptavidin optimized at 1 ug/mL. The linear regression equation of the reference curve was Y = 53.35 × X + 10294, and the *R*^2^ values were 0.9666 and 0.9565, respectively (Figure 7A,B), indicating that the linear relationship was relatively good. Additionally, the coefficient of variation CV (%) was 1.5184~11.9649%, which was less than 20%. Using the antigen HPV16 E7 oncoprotein content (ng) as the abscissa and the S/N ratio as the ordinate, it was found that the S/N was greater than 3 when the antigen was at 25 ng and above, indicating that the detection limit was lower than 25 ng (Figure 7C). Using the content of the antigen HPV16 E7 oncoprotein at 0 and 25 ng as the abscissa and the chemiluminescence value as the ordinate, the regression equation to calculate the detection limit was Y = 363.1 × X + 3441, *R*^2^ = 0.9823 (Figure 7D) and the chemiluminescence value = 3 × 3441 = 10324 when the S/N = 3, and detection limit = (10324 − 3441)/363.1=18.9562 ng. The CV were all less than 20%, and the detection limit was also the lowest limit of quantitative detection, indicating that the detection limit was 6.48 folds than that of the conventional double-antibody sandwich ELISA method for quantitative detection of HPV16 E7 oncoprotein.

This method was then used to detect the presence of HPV16 E7 oncoprotein in 20 μg total protein extracted from normal or neoplasmic cervical tissues. Bringing the chemiluminescence value of the specimens into the equation Y = 53.35 × X + 10294 to obtain the HPV16 E7 protein contents of cervical tissues, the result showed that the contents were all less than the detection limit (18.9562 ng). However, the HPV16 E7 protein content of twelve HPV16-positive cervical cancer tissues fluctuated from 102.1743 to 438.2473 ng, with S/N values > 3.0 (Figure 7E). Compared with that of the normal tissues and HPV52-positive cervical cancer tissues, the HPV16 E7 protein content of HPV16-positive cervical cancers was significantly positive (*p* < 0.01). These results showed that the chemiluminescence immunoassay method established was preliminarily effective for quantitively detecting HPV16 E7 oncoprotein in HPV16-positive cervical cancer tissues.

## 4. Discussion

The integration of HPV is an important factor during the malignant transformation of cervical epithelial cells [17,18]. HPV E6 and E7 are both oncogenic proteins [19], but the HPV E7 oncogenic protein is more critical [20]. Therefore, detection of HPV E7 is essential for the diagnosis of cervical cancer in the early stage [21]. Recently, the HPV18 L1  ELISA detecting method and HPV16 E7-binding affibody molecules and nanobodies against the HPV16 E7 oncoprotein have been shown to have promising potential for the diagnosis of HPV-induced cancers [22,23,24]. The goal of this research is to establish a method with high sensitivity for quantitative detection of the HPV16 E7 oncoprotein in cervical cancer tissues.

In this study, HPV16 E7-HIS fusion oncoprotein was successfully prepared, and the mAbs of mouse anti-HPV16 E7-HIS fusion oncoprotein were prepared and identified. The pair of mAbs with the strongest signals were used to establish a conventional double-antibody sandwich ELISA method and an LSAB-lumino-dual-antibody sandwich ELISA chemiluminescence immunoassay method. The results showed that the chemiluminescence immunoassay method was more sensitive than the conventional dual-antibody sandwich ELISA method in the quantitative detection of HPV16 E7-HIS fusion oncogenic protein. The clinical validity of this chemiluminescence immunoassay method was preliminarily verified with HPV16-positive cervical cancer specimens.

The reasons of why the established chemiluminescence immunoassay method based on LSAB-ELISA and luminol is better than the double-antibody sandwich ELISA based on HRP-labeled mAb are as follows: in the conventional double-antibody sandwich ELISA method, HRP directly labels the mAb without amplification, and the enzyme HRP itself is not sensitive, unstable, which causes the conventional double-antibody sandwich ELISA to have high background OD value and unsatisfactory sensitivity [25]. The LSAB-ELISA is based on the principle of conventional ELISA and uses the height between biotin and streptavidin to amplify the effect and then replace the chromogenic substrate TMB with the chemiluminescence substrate luminol [26,27]. Additionally, dozens of biotin molecules are coupled to one antibody molecule without affecting the biological activity of antibody 69E2. The antigen, HPV16 E7-HIS fusion oncoprotein, binds to biotinylated mAb 69E2. Streptavidin in HRP-Streptavidin binds to four biotins in biotinylated antibodies Biotin-69E2. A streptavidin can bind multiple HRP enzymes, which catalyze chemiluminescent substrate luminol, thereby making the sensitivity of the chemiluminescent substrate higher than that of the chromogenic substrate, and the signal is amplified in multiple layers [28,29]. This method may be used to trace the antigen, antibodies, and receptors quantitatively and qualitatively.

The detection limit for the HPV16 E7 protein detected by the chemiluminescence immunoassay based on the LSAB-lumino-dual-antibody sandwich ELISA established in this study was 18.95 ng, which was 6.48 fold different from the detection limit of 122.92 ng of the dual-antibody sandwich ELISA method, indicating that the sensitivity of the luminescent immunoassay was higher than that of ordinary double-antibody sandwich ELISA. The S/N ratio of the HPV16 E7 protein content of the HPV16-positive cervical cancer tissues detected by the chemiluminescence immunoassay based on LSAB-ELISA-lumino-ELISA established in this subject was greater than 10, indicating that the signal was reliable and can also be detected. The S/N ratio of HPV16 E7 protein in the adjacent tissues was greater than 6.0, indicating that the method was reliable and effective, and provides some data references for further quantitative research.

To detect HPV16 E7 oncoproteins, corresponding anti-HPV16 E7 oncoprotein mAbs are required. To prepare the mAbs, the antigen HPV16 E7 oncoprotein must be successfully prepared. HPV cannot be cultivated outside of a host cell in a laboratory [30]. Therefore, HPV16 E7 can only be prepared by genetic engineering techniques. Munger K. et al. showed that the HPV E7 protein often performs its function in the form of dimers or multimers in the zinc binding region [31]. The molecular weight of the HPV16 E7 oncoprotein monomer is 12 kDa [32], and the molecular weight of the HPV16 E7-HIS fusion oncoprotein prepared in this study was about 15–18 kDa, which may be caused by the protein’s negative charge [14]. Because the HPV16 E7-HIS fusion oncoproteins cannot be completely equivalent to the natural HPV16 E7 oncoproteins in cervical cancer cell lines and cervical cancer tissues, the effectiveness and specificity of the mAbs prepared using the HPV16 E7-HIS fusion oncoproteins as antigens needed to be identified. Therefore, we used ICC, IF, IHC, Western blot, and the LSAB-ELISA reactions of cervical cancer tissues to verify the effectiveness and specificity of the mAbs against the HPV16 E7 protein.

The successful detection of HPV16 E7 oncoprotein greatly depends on successful mAb pairing. The most successful aspect of this study has been obtaining a pair of mAbs that can be paired and have a strong signal. The capture antibody is an IgM subtype and the detection antibody is an IgG2a subtype. The combination of the mAb with the capture antibody as an IgM subtype and the detection antibody as an IgG2a subtype is the biggest innovation of this study. The reasons for the successful analysis of the pairing are as follows: (1) The IgM mAb 79A11 of the paired mAbs was derived from the spleen cells of an immunized mouse, and the IgG2a mAb of the paired mAb was also derived from the same immunized mouse lymph node cells, which confirmed that the two mAbs are directed against two different epitopes in the same antigen HPV16 E7-HIS fusion oncoprotein. (2) Although the affinity of IgG and an antigen is stronger than IgM, as a pentamer, an IgM can bind to 10 antigens and can access cryptic antigens denied to IgG [33]. IgM was used as a capture antibody in the double-antibody sandwich ELISA with less steric hindrance, which was more suitable for capturing antigens [34]. (3) Further analysis determined that the mAb 79A11 subtype was indeed IgM, 69E2 was IgG2a, and the amino acid sequences of the base sequences of the light and heavy chains of the two mAbs were significantly different.

The specific epitopes of the mAbs 79A11 and 69E2 are very similar since they both contain three exposed peptides—HPV16 E7_49–66_, HPV16 E7_73–85_, and HPV16 E7_91–97_. According to the data showing that the mAb 79A11 could be paired with the mAb 69E2 with strong pairing signals, it was proven that the specific epitopes of the mAbs 79A11 and 69E2 were in two different monomers of the same HPV16 E7 protein dimer. Combined with the result of the mAb 79A11 affinity test showing that the binding of mAb 79A11 to the antigen HPV16 E7 protein was eluted by high salt, it was speculated that the specific epitope peptides of mAb 79A11 were likely to contain lysines. This speculation was also supported by two pieces of evidence: (1) In the 3D crystal structure modeling diagram of the HPV16 E7 protein dimer, the 60th amino acid and the 97th amino acid of the HPV16E7 protein were lysines, and the positions of K60 and K97 were exposed and adjacent in space. (2) Indirect ELISA and indirect competition ELISA also showed that the mAb 79A11 specifically bound to the overlapping peptides HPV16 E7_49–66_ and HPV16 E7_85–98_, which contained K60 and K97, respectively. In contrast, the epitope of mAb 69E2 was less likely to contain lysine, because the affinity of mAb 69E2 to HPV16E7 protein was very high (5.60 × 10^−9^).

The detection method in this study can also optimize certain conditions to increase its sensitivity, such as using fresh antigen antibodies or obtaining the optimal conditions to prepare the reference curve in the fastest time. A better detection system is also needed to increase its sensitivity and reduce the background OD value. For example, HRP-Streptavidin can be replaced with alkaline phosphatase (AP)-Streptavidin [35]. Because the sensitivity of AP is higher than that of HRP, the background OD value is lower and the signal is more stable [36]. It is also possible to bind the capture antibody to magnetic beads. One limitation of this project is that the number of specimens is too small. In future research, more specimens should be used to verify the effectiveness of the method.

In conclusion, we successfully obtained two novel anti-HPV16 E7 oncoprotein mAbs and established an efficient method for the detection of anti-HPV16 E7 oncoprotein. The two novel anti-HPV16 E7 mAbs acquired in this study have many potential applications in experimental research and clinical diagnosis and therapy. Firstly, they can be developed as diagnostic kits, rapid diagnostic test strips, antibody-based biosensors or immunosensors, or other products that can detect the content of HPV16 E7 oncoprotein in cervical exfoliated cells, some of which may differentiate high-risk from low-risk [37,38,39]. Secondly, the anti-HPV16 E7 protein mAbs can be transformed into human/mouse chimeric antibodies, or humanized antibodies through Fc modifications, which can be used for blocking the carcinogenic effect induced by the E7 oncogenic protein [40,41,42]. Thirdly, HPV16 E7 human/mouse chimeric antibodies, or humanized HPV16 E7 antibodies can be conjugated with anti-cervical cancer drugs or radiolabels to develop radionuclide imaging agents for both the diagnosis and biological missiles for therapy of HPV16-positive neoplasms, including but not limited to, cervical cancers [43,44]. Fourthly, since the mAb MW is so large that it is difficult to be transported into cellular plasma, resulting in the mAb failing to block the intracellular E7 oncoprotein, the mAbs can provide some clues for the design of some intracellular antibodies (also known as single-chain variable fragments (scFvs)), which have been shown to have the potential for the treatment of HPV16-positive tumors [45,46,47,48]. Finally, these mAbs may also be used in experimental research. For instance, they can be used in immunoaffinity chromatography for purification of the HPV16 E7 antigen. Therefore, this study lies a foundation for the further exploration of antibody-based diagnosis and therapy for cervical cancer.

## Figures and Tables

**Table 1:** Affinity measurement of HPV16 E7 to 69E2.

Ligand	Analyte	Chi² (RU²)	ka (1/Ms)	kd (1/s)	KD (M)	Rmax (RU)
HPV16 E7	69E2	0.339	1.98 × 10^4^	1.11 × 10^−4^	5.60 × 10^−9^	155.2

## Supplementary Materials

Supplementary materials can be found at https://www.mdpi.com/1999-4915/12/3/333/s1.
