# Supplementary material for: Preparation, Characterization and Diagnostic Valuation of Two Novel Anti-HPV16 E7 Oncoprotein Monoclonal Antibodies
Source: Viruses. 2020 Mar 19;12(3):333. doi: 10.3390/v12030333 (PMC7150828; doi:10.3390/v12030333)
Supplement: Supplementary file 1 [file viruses-12-00333-s001.zip › Supplementary Materials S1.docx]

**Appendix A**

**Amino acid sequences of 69E2 and 79A11 anti-HPV16E7 protein antibodies.**

(1) Heavy chain of antibody 69E2: Amino acids sequence (468 aa).

Leader sequence-FR1-CDR1-FR2-CDR2-FR3-CDR3-FR4-Constant region-Stop codon

MGWSCIIFFLVATATGVHSQVQLQQSGPEVVRPGVSVKISCKGSGYTFTDYAMHWVKQSHAKSLEWIGVISTYNGNTNYNQKFKGKATVTVDKSSSTAYMELARLTSEDSAIYYCARRGFGNSWFAYWGQGTLVTVSAAKTTAPSVYPLAPVCGDTTGSSVTLGCLVKGYFPEPVTLTWNSGSLSSGVHTFPAVLQSDLYTLSSSVTVTSSTWPSQSITCNVAHPASSTKVDKKIEPRGPTIKPCPPCKCPAPNLLGGPSVFIFPPKIKDVLMISLSPIVTCVVVDVSEDDPDVQISWFVNNVEVHTAQTQTHREDYNSTLRVVSALPIQHQDWMSGKEFKCKVNNKDLPAPIERTISKPKGSVRAPQVYVLPPPEEEMTKKQVTLTCMVTDFMPEDIYVEWTNNGKTELNYKNTEPVLDSDGSYFMYSKLRVEKKNWVERNSYSCSVVHEGLHNHHTTKSFSRTPGK-

(2) Light chain of antibody 69E2: Amino acids sequence (235 aa).

Leader sequence-FR1-CDR1-FR2-CDR2-FR3-CDR3-FR4-Constant region-Stop codon

MDFQVQIFSFLLISASVMMSRGENVLTQSPAIMSASPGGKVTMTCSASSGVSYMHWYQQKSSTSPKLWIYDTSNLASGVPGRFSGSGSGNSYSLTISSMEAEDVATYYCFQGSGYPFTFGSGTKLEIRRADAAPTVSIFPPSSEQLTSGGASVVCFLNNFYPKDINVKWKIDGSERQNGVLNSWTDQDSKDSTYSMSSTLTLTKDEYERHNSYTCEATHKTSTSPIVKSFNRNEC-

(**3**) Heavy chain of antibody 79A11: Amino acids sequence (592 aa).

Leader sequence-FR1-CDR1-FR2-CDR2-FR3-CDR3-FR4-Constant region-Stop codon

MLLGLKWVFFVVFYQGVHCEVQLVESGGGLVQPKGSLKLSCAASGFTFNTYAMNWVRQAPGKGLEWVARIRSKSKNYATYYAESVKDRFTISRDDSQSMLYLQMNNLKTEDTAMYHCVRGYRYLDYWGQGTSVTVSSESQSFPNVFPLVSCESPLSDKNLVAMGCLARDFLPSTISFTWNYQNNTEVIQGIRTFPTLRTGGKYLATSQVLLSPKSILEGSDEYLVCKIHYGGKNRDLHVPIPAVAEMNPNVNVFVPPRDGFSGPAPRKSKLICEATNFTPKPITVSWLKDGKLVESGFTTDPVTIENKGSTPQTYKVISTLTISEIDWLNLNVYTCRVDHRGLTFLKNVSSTCAASPSTDILTFTIPPSFADIFLSKSANLTCLVSNLATYETLNISWASQSGEPLETKIKIMESHPNGTFSAKGVASVCVEDWNNRKEFVCTVTHRDLPSPQKKFISKPNEVHKHPPAVYLLPPAREQLNLRESATVTCLVKGFSPADISVQWLQRGQLLPQEKYVTSAPMPEPGAPGFYFTHSILTVTEEEWNSGETYTCVVGHEALPHLVTERTVDKSTGKPTLYNVSLIMSDTGGTCY-

(**4**) Light chain of antibody 79A11: Amino acids sequence (239 aa).

Leader sequence-FR1-CDR1-FR2-CDR2-FR3-CDR3-FR4-Constant region-Stop codon

MMSPAQFLFLLVLWIRETNGDVVMTQTPLTLSVTIGQPASISCKSSQSLLDSDGKTYLNWLLQRPGQSPKRLIYLVSKLDSGVPDRFTGSGSGTDFTLKISRVEAEDLGVYYCWQGTHFPWTFGGGTKLELKRADAAPTVSIFPPSSEQLTSGGASVVCFLNNFYPKDINVKWKIDGSERQNGVLNSWTDQDSKDSTYSMSSTLTLTKDEYERHNSYTCEATHKTSTSPIVKSFNRNEC-
