# Supplementary material for: Preparation, Characterization and Diagnostic Valuation of Two Novel Anti-HPV16 E7 Oncoprotein Monoclonal Antibodies
Source: Viruses. 2020 Mar 19;12(3):333. doi: 10.3390/v12030333 (PMC7150828; doi:10.3390/v12030333)
Supplement: Supplementary file 1 [file viruses-12-00333-s001.zip › Supplementary Materials S2.docx]

**Appendix B**

**Supplementary figures**


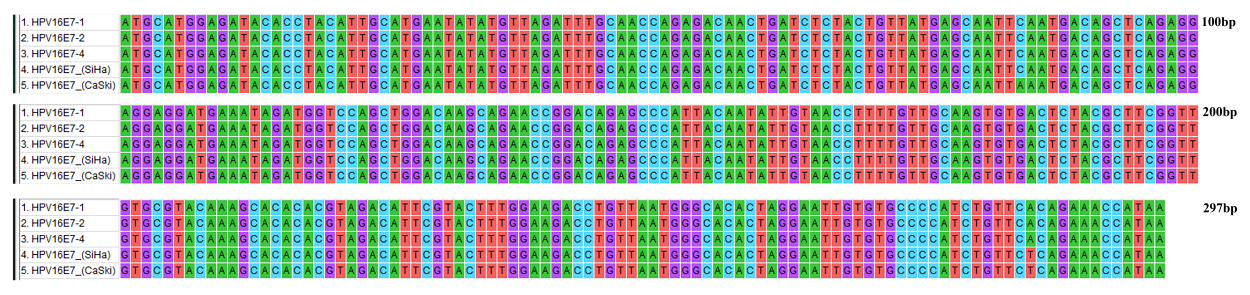


**Figure S1.** Sequence comparison of HPV16 E7 gene in recombinant plasmid pET-28a (+) - HPV16 E7 with HPV16 E7 genes in CaSki and SiHa cells


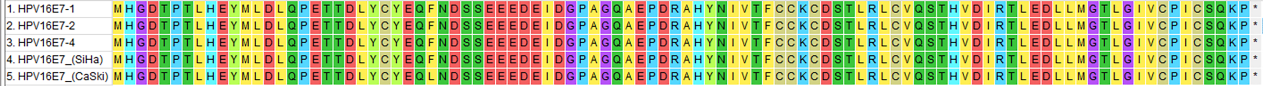


**Figure S2.** Sequence comparison of HPV16 E7 amino acids in three recombinant plasmid pET-28a (+) - HPV16 E7 with HPV16 E7 amino acids in CaSki and SiHa cells

**
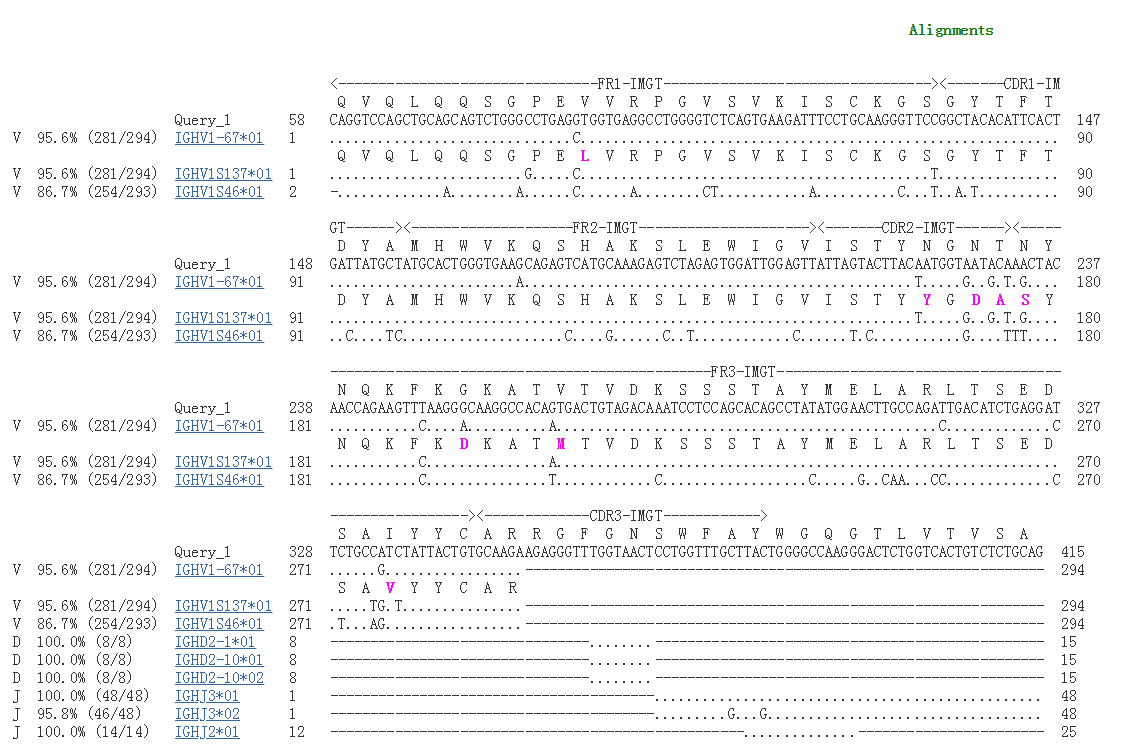
**

**Figure S3.** Alignment of sequences of heavy chain of 69E2 anti-HPV16 E7 protein antibody to that of other Ig sequences in the IgBLAST database


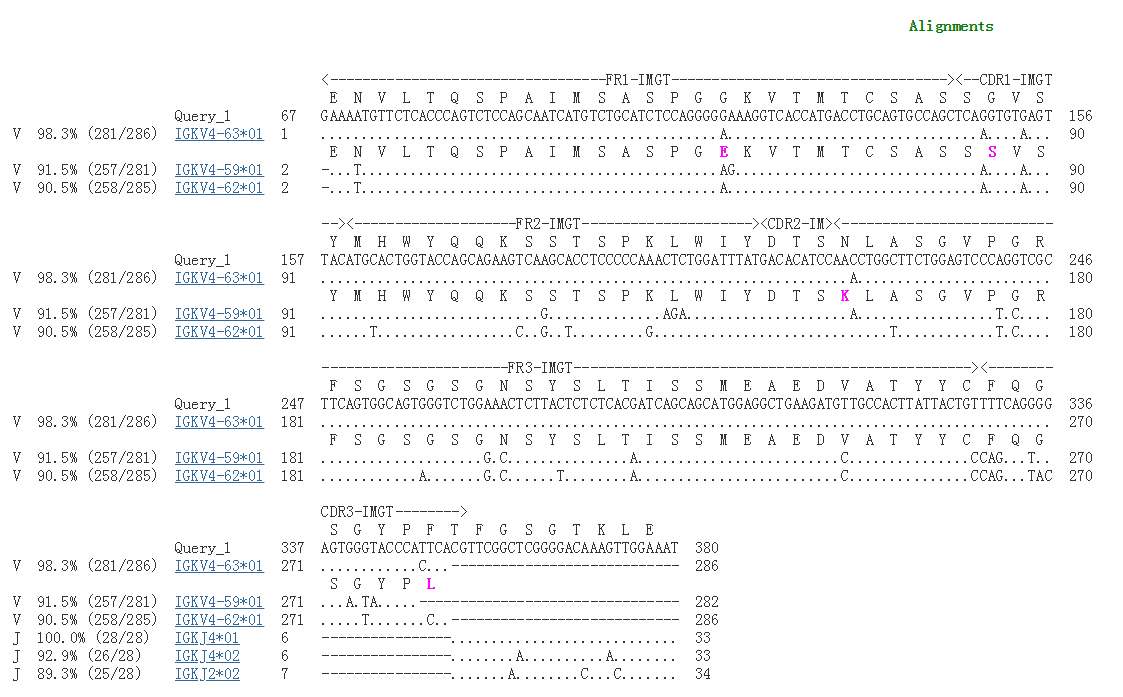


**Figure S4.** Alignment of sequences of light chain of 69E2 anti-HPV16 E7 protein antibody to that of other Ig sequences in the IgBLAST database.


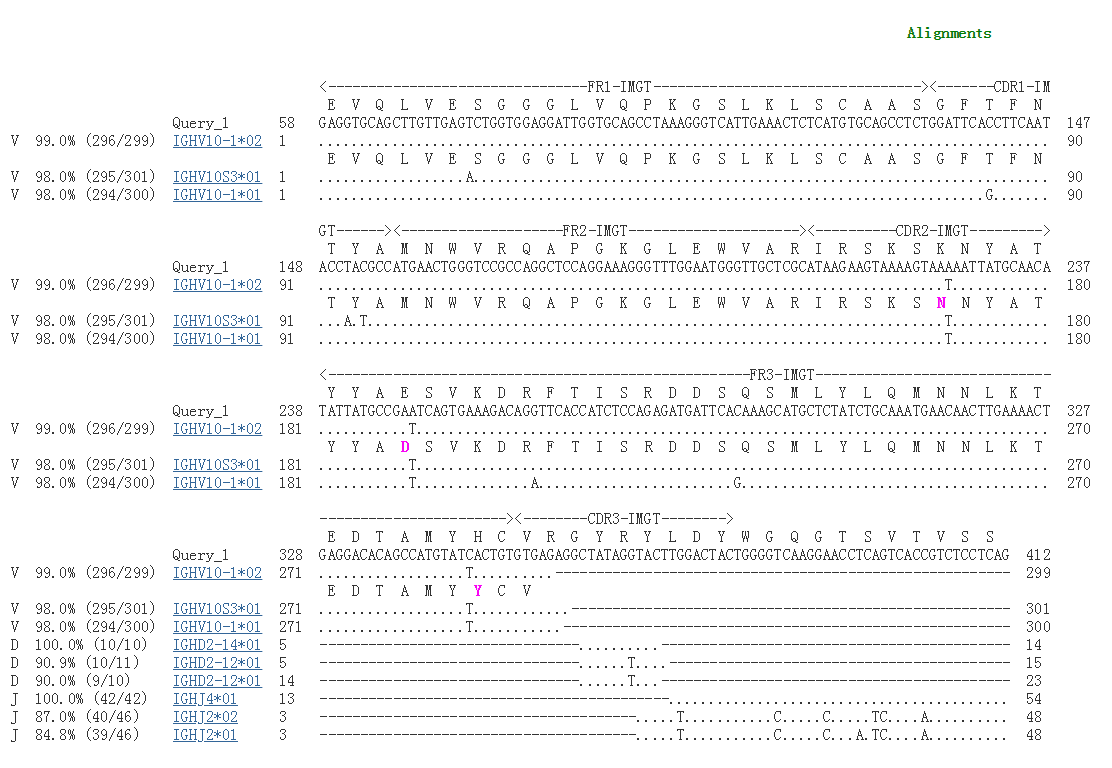


**Figure S5.** Alignment of sequences of heavy chain of 79A11 anti-HPV16 E7 protein antibody to that of other Ig sequences in the IgBLAST database.


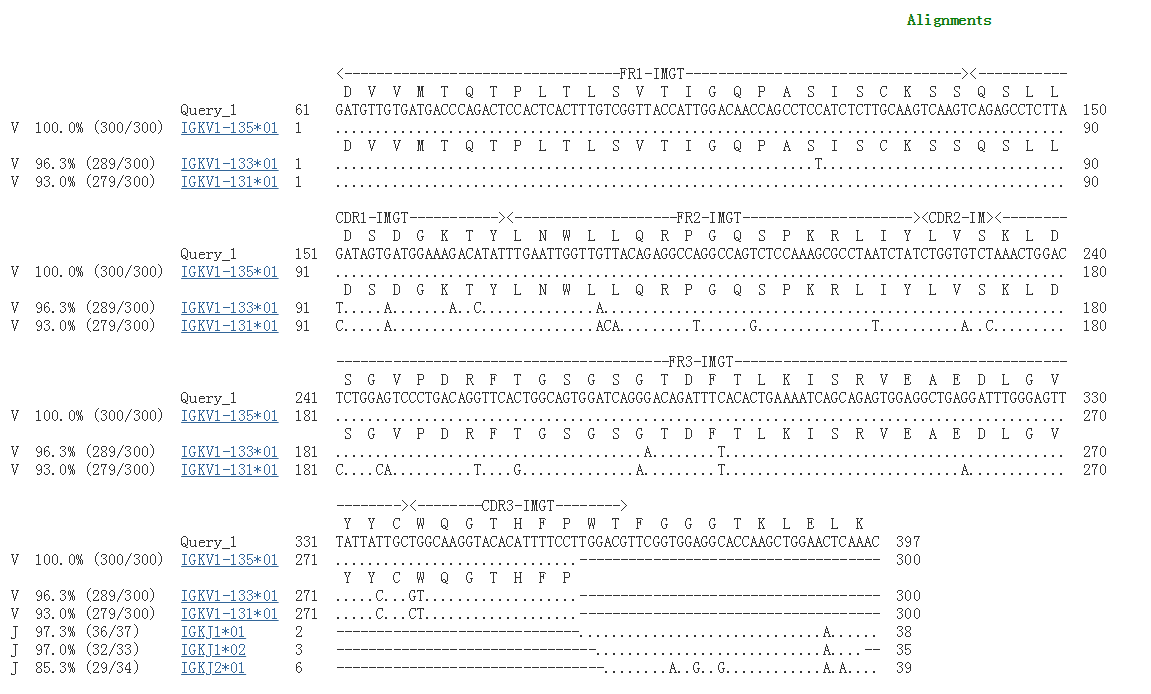


**Figure S6.** Alignment of sequences of light chain of 79A11 anti-HPV16 E7 protein antibody to that of other Ig sequences in the IgBLAST database.


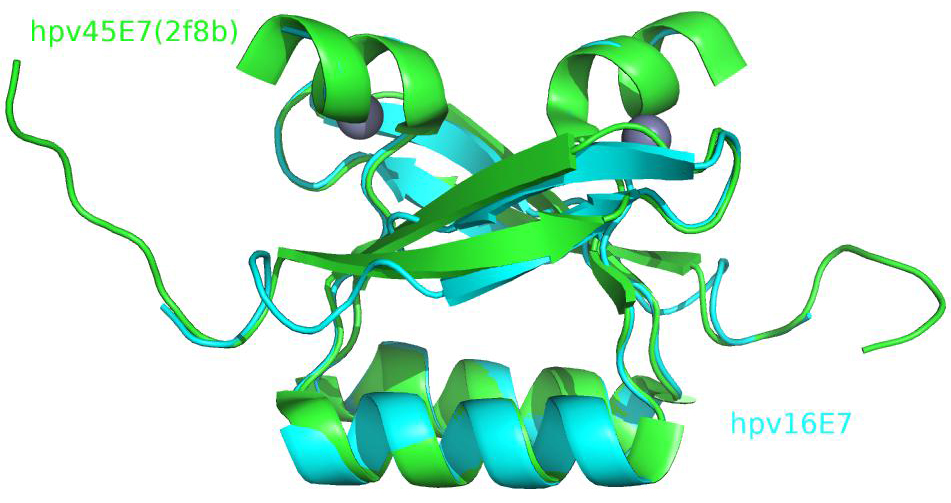


**Figure S7.** Comparison of HPV16 E7 and HPV45 E7 protein crystal structure modeling diagram.
